# Supplementary material for: Patterns of Intron Gain and Loss in Fungi
Source: PLoS Biol. 2004 Nov 30;2(12):e422. doi: 10.1371/journal.pbio.0020422 (PMC532390; doi:10.1371/journal.pbio.0020422)
Supplement: Table S1 — Also available at http://genes.mit.edu/NielsenEtAl/. (4.3 MB ZIP). [file pbio.0020422.st001.zip › NielsenEtAl/html/629.html]

AN5355.1.NCU08402.1.MG00246.1.FG01686.1


```
 CLUSTAL W (1.82) Multiple Sequence Alignments - Introns Inserted


Sequence 1: MG00246.1	364 aa
Sequence 2: FG01686.1	361 aa
Sequence 3: AN5355.1	359 aa
Sequence 4: NCU08402.1	368 aa
Alignment Length: 372 aa
Number Identitical Residues: 162 aa
Alignment Score (without introns) 8769


MG00246.1 	MAATDYKFEGWLGDSPESANGKMRWGEFEPKKWTEDDVDIKISHCGICGSDLHTLSSGW~
NCU08402.1	MSGTDYKFEGWLGFDKSSAHGNMQWGEFEPKPWEETDIDIKIECCGVCGSDIHTLRSGW0
FG01686.1 	--MAEYEFKGWLGKDAESANGKMEWGSFEPKKWEENDVDIKITHCGVCGSDLHTLRSGW~
AN5355.1  	-MSTDYKFEGWLGLDAGSVDGKMQWKEFEPKPWEETDVDIKISHCGICGSDLHTLRSGW0
          	   ::*:*:**** .  *..*:*.* .**** * * *:****  **:****:*** *** 

MG00246.1 	GPTPYP1CCVGHEIVGRAVRVGSNVKD-IKVGDRVGVGAQARSCMS---ADCPECSIGRE
NCU08402.1	GPTNYP~CCVGHEVVGTVVRAGSKVEGGIKVGDRVGVGAQSDACRNRTDKHCKICADGNY
FG01686.1 	GETPFP1CCVGHEIVGKAVKVGKNVKN-IKVGDRVGVGAQARSCLR---EDCIECSAGRV
AN5355.1  	GPTNYP~CCVGHEIVGTAVRVGSQVKH-IKVGDRVGVGAQSESCVGR-KGECNECATSNE
          	* * :* ******:** .*:.*.:*:  ************: :*    . .*  *: .. 

MG00246.1 	NYCSTKGQLINTYGSVYPGDEGKSYGGYATYNRTNAKFCIKIPDGVPSEYAAPLMCGGVT
NCU08402.1	NYCRV--HHVGTYNGVYMNG-GKSYGGYATYQRVPSFFAVKIPESMPSSHAAPMLCAGVT
FG01686.1 	NYCDN---MVSTYGSVYPDGIGKSYGGYADYNRTDSRFVVKIPEALPSELAAPMLCGGVT
AN5355.1  	PYCTK--HFAGTYNGVFMNG-GKSYGGYALYNRSPAHFAIKIPDAIPSAHAAPMMCGGVT
          	 **       .**..*: .. ******** *:*  : * :***:.:**  ***::*.***

MG00246.1 	VWSPLKDNKCGPGKNVGIVGVGGLGHFAVLFAKAMGADKVVGVSRRESKRKEVMSLGADD
NCU08402.1	TYAPLKFHGVGPGKRVGVIGLGGLGHFGVMWAKALGADEVVVISRSSSKKADAMAMGATG
FG01686.1 	VYAPLRNNGCGPGKTVGIVGVGGLGHFGILFAKALGADKVVGISRKASKRDEVLALGADS
AN5355.1  	VYSPLKHYGCGPGKTVGIIGIGGLGHFGVLFAKALGADRVVAISRKSDKRADALKLGADE
          	.::**:    **** **::*:******.:::***:***.** :**  .*: :.: :**  

MG00246.1 	YIATEDEKDWEKHHRRSLDLIICTVSHTGMPLDKYLMLLKPNGILINVGVPEGGALPNLN
NCU08402.1	FIATGEDPEWLNKNRSSLDIILCTASSADMPFNDYLFLLDLDGVFIQVGAPDD-DLPRIK
FG01686.1 	YIATDDDEDWANKYAKTVDIIVCTVSSSKMPFSDYFKLLRHGGNFIQVGAPDSGLLPPVN
AN5355.1  	YVATAEDTEWMANNKRSLDLIVCTVSSSKMPINDYVSLLKVDGTMIQVGAPEDGALT-IP
          	::** :: :*  :   ::*:*:**.* : **:..*. **  .* :*:**.*:.. *. : 

MG00246.1 	AFTLVINAVSVGGSLIGSPSEIREMLEFVAEHNIKPLIETRPMKEANQAILDMEHGKARY
NCU08402.1	QMALIFNRIKLTGSLIGSPDDIREMFQLAVDKKVQPWISEIPMKEANKAIVDFEDGKPRY
FG01686.1 	AFTLIKGGFKLSGSLIGSPADIEEMLELAVKKNVKPWIEKRPMEDANQTIIDIEEGKARY
AN5355.1  	AFTLIANRVKLGGSLIGSPDTIREMLELAAEKGVKPWVEERPMKDANQAIKDMDAGDARY
          	 ::*: . ..: *******  *.**:::...: ::* :.  **::**::* *:: *..**

MG00246.1 	RYVLVNEDNE----
NCU08402.1	RFVLKNEDYTPVQA
FG01686.1 	RYVLVNQKNIEQ--
AN5355.1  	RYVLVNDH------
          	*:** *:.
```
